# Evolutionary history of the medaka *long-wavelength sensitive* genes and effects of artificial regression by gene loss on behavioural photosensitivity

**DOI:** 10.1038/s41598-019-39978-6

**Published:** 2019-02-25

**Authors:** Yumi Harada, Megumi Matsuo, Yasuhiro Kamei, Mayuko Goto, Shoji Fukamachi

**Affiliations:** 1 Laboratory of Evolutionary Genetics, Department of Chemical and Biological Sciences, Japan Women’s University, Tokyo, Japan; 2 Spectrography and Bioimaging Facility, Core Research Facilities, National Institute for Basic Biology, Aichi, Japan; 3 School of Life Science, The Graduate University for Advanced Studies (SOKENDAI), Kanagawa, Japan

## Abstract

Tandem gene duplication has led to an expansion of cone-opsin repertoires in many fish, but the resulting functional advantages have only been conjectured without empirical demonstration. Medaka (*Oryzias latipes* and *O. sakaizumii*) have eight (two *red*, three *green*, two *blue*, and one *violet*) *cone opsin* genes. Absorbance maxima (λ_max_) of the proteins vary from 356 nm to 562 nm, but those of the red opsins (long-wavelength sensitive; LWS) are nearly identical, obscuring the necessity of their coexistence. Here, we compared the *LWSa* and *LWSb* loci of these sister species and found that the gene duplication occurred long before the *latipes*–*sakaizumii* speciation (4–18 million years ago), and the high sequence similarity between the paralogues is the result of at least two events of gene conversion. These repetitive gene conversions would indicate the importance for medaka of retaining two identical *LWS*s in the genome. However, a newly established medaka mutant with a single *LWS* showed no defect in *LWS* expression or behavioural red-light sensitivity, demonstrating functional redundancy of the paralogs. Thus, as with many other genes after whole-genome duplication, the redundant *LWS* might be on the way to being lost from the current *cone opsin* repertoire. Thus, non-allelic gene conversion may temporarily provide an easier and more frequent solution than gene loss for reducing genetic diversity, which should be considered when assessing history of gene evolution by phylogenetic analyses.

## Introduction

Under daylight, vertebrates predominantly receive light by cone cells in the retina. The cones express light-absorbing proteins, the cone opsins, which are classified into four types: red (long-wavelength sensitive; LWS), green (rhodopsin 2; RH2), blue (short-wavelength sensitive 2; SWS2), and violet (SWS1). Vertebrates have various cone opsin repertoires (e.g., birds and fish have all types, whereas mammals [except for monotremes] have only LWS and SWS1^1^), which may reflect visual adaptation in each habitat.

Many fish have increased the number of *cone opsin* genes by tandem (and whole-genome) duplication and the repertoire often consists of eight or more subtypes^2–4^. The repertoires have been extensively studied in a macro scale, which revealed repetitive gene gains and losses during evolutionary radiation of teleosts^2,4^. This diversification of *cone opsin* repertoires is often discussed in relation to various underwater light conditions (e.g., depth, turbidity, or colour of mates^5–7^), but direct empirical evidence supporting these speculations (i.e., what behavioural/ecological advantages the repertoires actually provide) is lacking.

The functional advantage of having two paralogous LWSs (often referred to as LWS and medium-wavelength sensitive [MWS]) is obvious in humans, because a loss of either paralogues causes the red–green colour-blindness^8^. Similarly, for some monkeys, trichromats have more opportunities for foraging ripe fruits or flowers in woods than dichromats^9–11^. To our knowledge, these are the only examples in which advantages for having cone opsin subtypes have been demonstrated at a behavioural/ecological level.

Medaka, *Oryzias latipes* and *O. sakaizumii*, are small freshwater fish native to the Far East. The genus comprises 32 species^12^ that live in variable habitats ranging from freshwater to brackish water in East/South Asia. Despite their distinctive morphological differences^13^, *O. latipes* and *O. sakaizumii* have been considered to be allopatric subpopulations of the same species until recently (i.e., the Southern and Northern populations of *O. latipes*, respectively). Their genomic sequences are highly polymorphic (e.g., an SNP rate of 3.42%^14^), but the species are sexually compatible, which has been one of the best features of this model animal for genetic/genomic experiments such as locus mapping and positional cloning^15,16^. Although other species in the genus *Oryzias* are also used for research (e.g., the sex-determining gene^17^), most studies are performed using these youngest sister species (sometimes without discrimination).

Medaka have two *LWS* paralogues as in humans. However, unlike the human LWSs, proteins encoded by the medaka *LWSa* and *LWSb* are 98.3% (351/357) identical and their absorbance maxima (λ_max_) are essentially the same at 561 ± 1 and 562 ± 2 nm (mean ± standard error of the mean [sem]), respectively^18^. This seems to be an exceptional case, considering that the LWS paralogues of other vertebrates (e.g., catarrhine primates, zebrafish, and guppy^19–21^) exhibit mutually distinct λ_max_. Therefore, it seems to be that either (1) duplication of the medaka *LWS* occurred very recently, or (2) diversification (neo-functionalization) of the *LWS* paralogues since an ancient duplication has been suppressed by a mechanism such as reduced mutation rate or gene conversion^22^.

In this study, we examined nucleotide sequences of the *LWSa* and *LWSb* loci of medaka to investigate their evolutionary history. In addition, we established a medaka strain with a single *LWS* using the CRISPR/Cas9 system and assessed whether the fish has a functional disadvantage because of the decreased copy number^23^.

## Results

### Orthologous comparison of the *LWS* loci between *O. latipes* and *O. sakaizumii*

We verified that genomic sequences of *LWSa* and *LWSb* of *O. sakaizumii* (the HNI strain) registered in the GenBank database (a total of 4,320 bp) were perfectly identical to the whole-genome sequence recently available at the UTGB database (http://utgenome.org/medaka_v2/#!Top.md), except for a single A–G mismatch at the 3′ UTR of *LWSa* (data not shown). We also obtained a corresponding genomic sequence of *O. latipes* (the Hd-rR strain) from the UTGB database. These genomic sequences (namely, about a 20-kb region between the upstream-neighbouring *SWS2b* and downstream-neighbouring *gnl3-like* loci) contained no gap or ambiguous nucleotide. There was no protein-coding gene other than *LWSa* and *LWSb* in this region according to the BLASTX program (although at least one *microRNA* gene does exist^24^).

**Figure 1:**
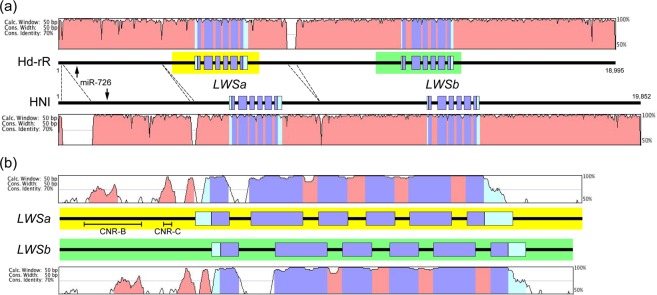
The medaka *LWSa* and *LWSb* loci. (**a**) Orthologous comparison between *O. latipes* (the Hd-rR inbred strain) and *O. sakaizumii* (the HNI inbred strain). Outputs of the mVISTA program using genomic sequences covering the *LWSa* and *LWSb* loci are shown at the top and bottom. The two bold black horizontal lines in the middle represent the genomic sequences, and boxes show the positions of exons: light blue, UTR; purple, coding region. Dotted lines indicate relatively large insertions/deletions. The positions of the *microRNA* (*miR-726*) gene, which is co-expressed with *LWSa*^24^, are shown by arrows. The regions highlighted in yellow and green were used for paralogous comparison in (b). (**b**) Paralogous comparison of *LWSa* and *LWSb* in *O. latipes*. The mVISTA outputs are shown at the top and bottom. The coding region, the 5′/3′ UTRs, a part of the 5′-upstream region, and the 2nd–5th introns are highly conserved. In spite of the conservation, the CNR-B and CNR-C sequences are shown to be dispensable for expressing *LWSa*^13^.

As shown in Fig. 1a, these sequences of the sister species were highly similar, except for a few relatively large insertions/deletions in the intergenic regions. In both species, the protein-coding region of *LWSa* and *LWSb* consists of 1,071 bp (excluding the stop codon) and is split into six exons with conserved exon–intron boundaries. There are 16 (14 synonymous and two non-synonymous) substitutions in *LWSa* between the species, whereas there are 10 (all synonymous) substitutions in *LWSb* (Table 1). Thus, the amino-acid sequences are 99.4% (355/357) and 100.0% (357/357) identical in LWSa and LWSb between the species, respectively.

**Table 1:** Number of nucleotide substitutions in the medaka *LWS* genes.

Length of the coding region in each exon of *LWSa* and *LWSb* (bp)			1st	2nd	3rd	4th	5th	6th	Total
			103	297	169	166	240	96	1,071
Orthologous comparison between *O. latipes* (Hd-rR) and *O. sakaizumii* (HNI)	in *LWSa*	Synonymous	0	5	3	3	2	1	14
		Non-synonymous	0	0	0	0	2	0	2
	in *LWSb*	Synonymous	0	5	1	2	2	0	10
		Non-synonymous	0	0	0	0	0	0	0
Paralogous comparison between *LWSa* and *LWSb*	in *O. latipes* (Hd-rR)	Synonymous	1	2	0	0	0	0	3
		Non-synonymous	2	2	0	0	0	0	4
	in *O. sakaizumii* (HNI)	Synonymous	1	2	2	1	0	1	7
		Non-synonymous	2	2	0	0	2	0	6

### Paralogous comparison of the *LWS* loci within *O. latipes* and *O. sakaizumii*

We then compared the *LWSa* and *LWSb* loci of *O. latipes* (Fig. 1b; a similar graphic was obtained from *O. sakaizumii* [data not shown], as expected from the high sequence similarity [Fig. 1a]) and found a few intriguing characteristics as explained below.

The coding sequences of *LWSa* and *LWSb* were 99.3% (1,064/1,071) identical, and the proteins were 98.9% (353/357) identical. However, the intergenic region was highly divergent, except for a few upstream regions, which may indicate a shared *cis*-regulatory mechanism between the paralogues^24^. In terms of the introns, the 1st and the rest (2nd–5th) showed a distinct difference: i.e., whereas the 1st intron was divergent (too different to be aligned as the intergenic regions), sequences of the 2nd–5th introns were 99.4% (351/353) identical between the paralogues (Fig. 1b).

A similar characteristic could be observed also in the coding region: i.e., all the seven nucleotide substitutions in the coding region (see above) were located on the 1st and 2nd exons (a total of 400 bp), whereas the 3rd–6th exons (a total of 671 bp) were 100% identical (Table 1). Thus, the 3′ region (from the 2nd intron to the 6th exon) was more similar between the paralogues than the 5′ region (from the 1st exon to the 2nd exon). This characteristic of the coding region was, however, not very clear in *O. sakaizumii*: i.e., among a total of 13 nucleotide substitutions, seven and six were located on the 1st–2nd and 3rd–6th exons, respectively (Table 1). The identity of the 2nd–5th introns also drops to 96.9% (344/355) with a 1-bp insertion/deletion.

Further comparison of the four nucleotide sequences (i.e., *LWSa* of *O. latipes*, *LWSb* of *O. latipes, LWSa* of *O. sakaizumii*, and *LWSb* of *O. sakaizumii*) revealed that the substitutions in the coding region can be classified into three types: (1) conserved between the orthologues (at five sites); (2) conserved between the paralogues (at eight sites); and (3) conserved between three of the four sequences (at 10 sites; Table 2). All the type (1) substitutions were found in the 1st exon and the 5′ half of the 2nd exon, whereas all the type (2) substitutions were found in the 3′ half of the 2nd exon and the downstream exons (Table 2). Furthermore, among a total of 18 substitutions in the highly conserved 2nd–5th introns (Fig. 1b), none and five sites belonged to the types (1) and (2) substitutions, respectively (Table 3).

**Table 2:** Number and types of nucleotide substitution in the medaka *LWS* genes (exons 1–6).

Substitution types^*^					Length of coding region in each exon (bp)						
	*O. latipes* (Hd-rR)		*O. sakaizumii* (HNI)		1st	2nd	3rd	4th	5th	6th	Total
	*LWSa*	*LWSb*	*LWSa*	*LWSb*	103	297	169	166	240	96	1,071
Conserved between orthologues	P	Q	P	Q	3	2	0	0	0	0	5
Conserved between paralogues	P	P	Q	Q	0	3	1	2	2	0	8
Others^**^					0	4	2	1	2	1	10

^*^Nucleotides (A, T, G, or C) are represented by P or Q.

^**^In all 10 cases, substitution occurred in one of the four sequences (eight and two substitutions in *LWSa* of HNI and *LWSb* of Hd-rR, respectively).

**Table 3:** Number and types of nucleotide substitution in the medaka *LWS* genes (introns 2–5).

Substitution types^*^					Length of the introns (bp)				
	*O. latipes* (Hd-rR)		*O. sakaizumii* (HNI)		2nd	3rd	4th	5th	Total
	*LWSa*	*LWSb*	*LWSa*	*LWSb*	85/87^***^	100/101^***^	82	86	353
Conserved between orthologues	P	Q	P	Q	0	0	0	0	0
Conserved between paralogues	P	P	Q	Q	1	1	2	1	5
Others^**^					4	6	2	1	13
(Inserted/deleted nucleotides)					**2**	1	0	0	3

^*^Nucleotides (A, T, G, or C) are represented by P or Q.

^**^In 12 of the 13 cases, substitution occurred in one of the four sequences. In one case, substitution occurred in two of the four sequences into different nucleotides.

^***^The lengths are not identical among the four sequences because of insertions/deletions.

**Figure 2:**
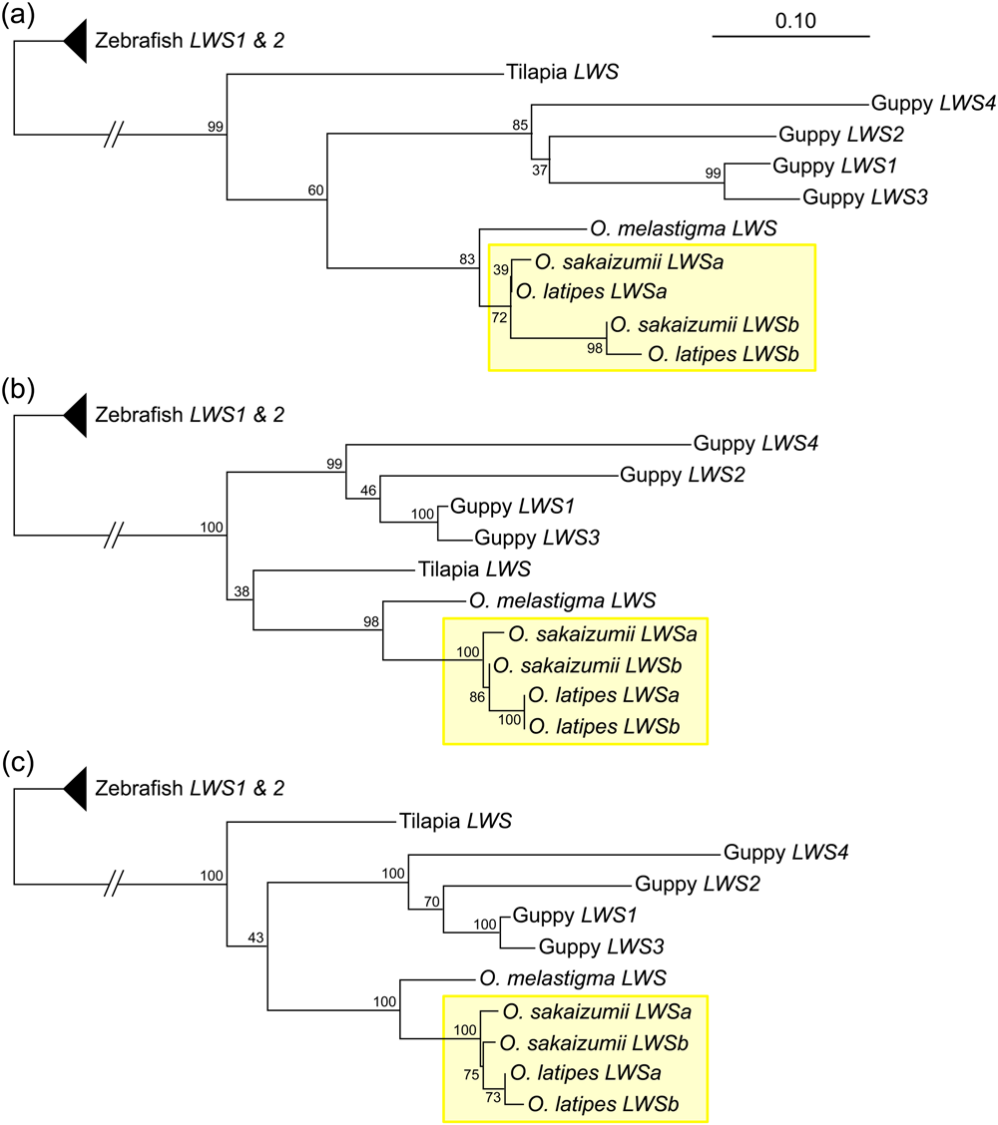
Phylogenetic relationships between the *LWS* genes of *O. latipes* and *O. sakaizumii*. (**a**) The 5′ part which might not undergo gene conversion (nucleotide positions 1–163), (**b**) the 3′ part which underwent gene conversion (297–1,071), or (**c**) the entire coding region (1–1,071) were used for the phylogenetic reconstruction by the maximum likelihood method (see Methods for details). Branch supports were shown as bootstrap values in 100 replications. The relationships focused in this study were highlighted by yellow boxes. The position of tilapia fluctuates for unknown reasons, but that of *O. melastigma* remains stable as an appropriate outgroup of medaka.

Taken together, using the middle of the 2nd exon as a border, the upstream region is more similar between the orthologues, whereas the downstream region is more similar between the paralogues in the *LWS* genes of medaka. This conclusion could be further supported by phylogenetic analyses (Fig. 2). The tree constructed using the upstream coding region (positions 1–163) supported sister groups of the orthologues (Fig. 2a), whereas the same analysis using the downstream coding region (positions 297–1,071) supported sister groups of the paralogues (Fig. 2b). We did not use nucleotides at positions 164–296 for these analyses, because the exact border between the upstream and downstream regions is unknown. The tree constructed using the entire coding region (Fig. 2c) supports the topology in Fig. 2b, likely because the downstream region (775 bp) is much longer than the upstream region (163 bp).

### Chimeric fusion of the *LWSa* and *LWSb*

**Figure 3:**
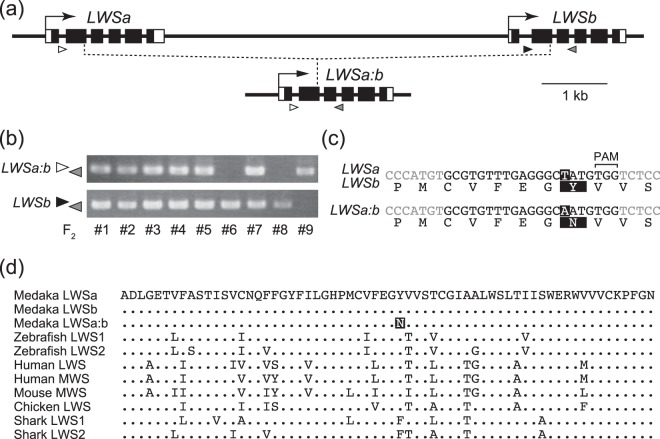
The *lws*^*a:b*^ mutant with a single *LWS*. (**a**) Structures of the *LWSa* and *LWSb* loci in the wild type (top) and the hybrid *LWSa:b* locus in the *lws*^*a:b*^ mutant (bottom). Black and white boxes indicate the coding region and 5′/3′ UTRs, respectively. The target sites of the gRNA exist in the 2nd exons, and the genomic region in-between (~7 kb) was deleted in the *lws*^*a:b*^ mutant (dotted lines). Arrowheads show approximate positions of the primers used for genotyping of F_2_ siblings: white, *LWSa*-specific forward; black, *LWSb*-specific forward; grey, *LWSb*-specific reverse (see the Methods for their sequences). (**b**) Genotyping of F_2_ siblings (#1~9) obtained by intercrossing heterozygous F_1_s. Three genotypes (i.e., wild type, heterozygote, and homozygote) could be distinguished by genomic PCR using two pairs of three primers (see the arrowheads in (**a**) for their positions). The white–grey pair amplifies a product (~0.6 kb) from the hybrid *LWSa:b* gene, whereas the black–grey pair amplifies a product from the wild-type *LWSb*. Thus, No. 1–5 and No. 7 are heterozygous, No. 6 and No. 8 are wild type, and No. 9 is homozygous. A full image of this gel is available as Supplementary Fig. S1. (**c**) The missense substitution in *LWSa:b*. The original sequences of *LWSa* and *LWSb* are shown at the top with translated amino acids. Black letters show the target sequence of the gRNA^6^. The sequence of *LWSa:b* is shown at the bottom. The substituted nucleotide (T > A) and amino acid (Y > N) are highlighted. (**d**) Comparison of LWSs among vertebrates. Amino-acid sequences were collected from the GenBank database and aligned. MWSs of human and mouse are paralogues of LWS. Amino acids identical to those of the medaka LWSa (top) are shown by dots. The substituted amino acid in *LWSa:b* is highlighted.

To assess the functional importance of having two nearly identical *LWS* genes for medaka, we established a medaka strain with a single *LWS* using the CRISPR/Cas9 system. Namely, we used gRNA targeting the 2nd exon of *LWSa* and *LWSb*^25^, anticipating that the double cleavages could cause a large deletion and an in-frame fusion of the paralogues (Fig. 3a).

Among 24 adult fish (G_0_) into which we had microinjected the gRNA and the *Cas9* mRNA^25^, nine possessed the hybrid gene in the caudal fin. From one of these G_0_s, we obtained 73 F_1_s, eight of which were heterozygous for the hybrid gene. We intercrossed the heterozygous F_1_s and obtained 28 F_2_ adults, whose genotype segregation ratio was wild type:heterozygous:homozygous = 8:16:4 (Fig. 3b; other data not shown). This ratio was not significantly different from that expected (7:14:7; *P* = 0.424, chi-squared test) and the homozygotes were indistinguishable from other siblings by appearance.

However, the hybrid gene (termed *LWSa:b*) had a missense substitution (from Y to N) within the target site (Fig. 3c). A mismatch between gRNA and its target site is known to reduce cleavage efficiency in the CRISPR/Cas9 system, particularly when the mismatch is close to the protospacer adjacent motif (PAM)^26^. Thus, this substitution located at 4-bp upstream of the PAM sequence should efficiently fix the *LWSa:b* in the G_0_ embryos, whereas other hybrid genes without such substitution would be repetitively cleaved until the gRNA or Cas9 protein lost their activity. Although we looked for hybrid genes without any substitution or with a silent substitution, none were found (the screening could not be completed because of accidental loss of other G_0_s and F_1_s during breeding).

The substituted tyrosine is widely, but not completely, conserved between vertebrates (Fig. 3d), which may indicate a replaceable role of this amino acid. The substituted tyrosine is the 128th residue from the N terminus (which corresponds to the 131st residue in the human LWS). This site does not correspond to any of “the tuning sites”^27^ that have been known so far to crucially affect λ_max_ (i.e., 180th, 197th, 277th, 285th, and 308th residues in the human LWS, which correspond to 164th, 181st, 261st, 269th, and 292nd residues in the bovine RH1).

### Transcription of *LWSa*, *LWSb*, and *LWSa:b*

**Figure 4:**
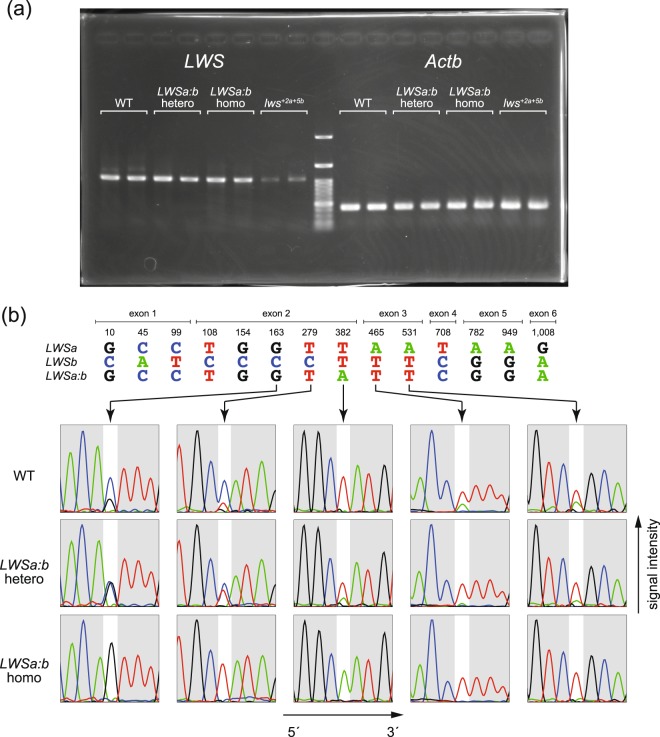
Expression of the *LWS* genes in the eyes of adult medaka. (**a**) Semi-quantitative reverse transcriptase PCR. Results of two fish from each genotype (wild type, *LWSa:b* heterozygote, *LWSa:b* homozygote, and *lws*^+*2a*+*5b*^ mutant^25^) are shown as representatives. The number of PCR cycles was 24 (the amplification was stopped before plateau; see Supplementary Fig. S2 for results of stepwise PCR) in both *LWS* and *β*-*actin* (*Actb*). Whereas the reduction in the *lws*^+*2a*+*5b*^ mutants, which has double frameshift mutations on *LWSa* and *LWSb*^25^, is apparent, expression in the *lws*^*a:b*^ mutants is equivalent to that in the wild type, despite the copy-number difference. (**b**) Direct sequencing of the reverse transcriptase PCR products. All the 14 nucleotide substitutions among the *LWS* genes (i.e., 13 substitutions between *LWSa* and *LWSb* [Table 1] and the missense substitution in *LWSa:b* [Fig. 3c]) are summarized at the top. The numbers indicate the positions of nucleotides from the translation-initiation site. Electropherograms at five sites (arrows) are shown as representatives. Note that (1) signals from *LWSb* are generally higher than those from *LWSa* in the wild type; (2) the homozygote expresses only *LWSa:b*; and (3) using the missense substitution at the position 382 as a border, signals from *LWSa* in the heterozygote are stronger or weaker than those in the wild type at the upstream or downstream region, respectively.

Genomic background of the host strains used in the above genetic engineering was *color interfere* (*ci*)^28,29^ (see the Methods section for more details about the strains). The *ci* might have originated from the *latipes*–*sakaizumii* hybrid population^30^, but its coding sequences of *LWSa* and *LWSb* were identical to those of *O. sakaizumii* (see Fig. 4b; other data not shown).

Using a pair of primers that commonly amplify the entire coding regions of *LWSa*, *LWSb*, and *LWSa:b*, we assessed expression of the mRNAs in the eyes of adult fish by semi-quantitative reverse transcription PCR. As shown in Fig. 4a, no apparent difference could be detected among the wild type, heterozygote, and homozygote, in spite of the copy-number difference. In terms of the *lws*^+*2a*+*5b*^ mutant, which has double frameshift mutations on both *LWSa* and *LWSb*^25^, the expression appears to be reduced, likely because of the nonsense-mediated mRNA decay (NMD).

Direct sequencing of these qPCR products revealed that the wild type expresses both *LWSa* and *LWSb*, with higher expression in *LWSb* (Fig. 4b, top). The homozygotes express only the hybrid *LWSa:b* (Fig. 4b, bottom). The signals (the peak intensity of electropherogram) from *LWSa* are stronger in the heterozygotes in comparison with those in the wild type at the upstream region from the CRISPR/Cas9 target site (e.g., the G and T signals at the positions 163 and 279), whereas the opposite is the case at the downstream region (e.g., the A signals at the positions 465 and 531; Fig. 4b, middle). This result could be explained by the *LWSa*:*LWSb* ratio in the heterozygote: i.e., the heterozygote has three copies of *LWS* (*LWSa*, *LWSb*, and *LWSa:b*), and the ratio is 2:1 and 1:2 at upstream and downstream of the target site, respectively.

### Spectral sensitivity of the *lws*^*a:b*^ mutant

**Figure 5:**
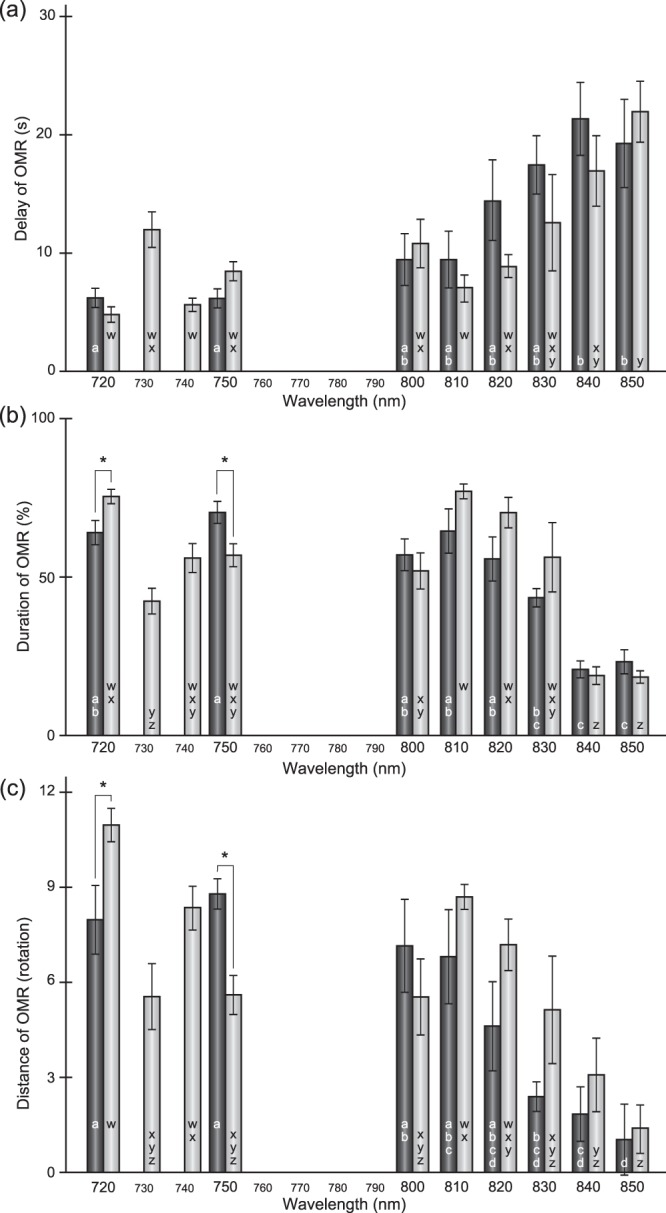
Optomotor response (OMR) of the *lws*^*a:b*^ mutant under monochromatic red light. The *lws*^*a:b*^ mutants (n = 6) were individually tested for OMR at every 10 nm in λ = 720–750 nm and 800–850 nm (light-grey bars). Dark-grey bars indicate the wild type (n = 5)^31^. Mean and standard error of the mean are shown. An asterisk indicates a significant difference between the wild type and mutant (*P* < 0.05; Student’s *t* test without correction). Different letters in the bars (a–d in the wild type and w–z in the mutant) indicate a significant difference according to a one-way ANOVA and the Tukey *post hoc* HSD test (*P* < 0.05). The OMR was evaluated by three parameters: (**a**) the average time (s) when fish started OMR (delay), (**b**) the average period (%) fish continued the OMR (duration), and (**c**) the total distance of swimming (round) towards the direction of the rotating stripes (distance). The fish occasionally showed no interest in the rotating stripes, which leads to values supporting low OMR (e.g., at λ = 730 nm of the *lws*^*a:b*^ mutants). However, such low OMR could be recovered at longer wavelength (e.g., λ = 810 nm), indicating that the low OMR was because of not decreased photosensitivity but decreased interests in the rotating stripes.

Lastly, we investigated behavioural red-light sensitivity of the homozygotes of *LWSa:b* (termed the *lws*^*a:b*^ mutant; n = 6) based on the optomotor response (OMR). At each wavelength we tested (i.e., 720 nm, 750 nm, and every 10 nm between 800 and 850 nm), the *lws*^*a:b*^ mutants responded to the rotating black-and-white stripes as quickly as the wild type (n = 5; Fig. 5a; *P* > 0.05, Student’s *t* test without correction). The duration of OMR (Fig. 5b) and a total distance of swimming (Fig. 5c) during the test were also similar between the wild type and the *lws*^*a:b*^ mutant at each wavelength, except for 720 nm and 750 nm (P < 0.05). Results of the OMR test are not always stable, because sometimes fish intentionally ignore or go against the moving stripes^31^. If it is the case, the OMR could be recovered at longer wavelengths (e.g., at λ = 810 nm; Fig. 5), which was an important criterion for judging whether fish do not or cannot see the stripes.

Significant attenuation of the OMR (i.e., gradual reduction in photosensitivity) could be detected in all three parameters described above (i.e., increase in the delay and decreases in the duration and distance) at λ ≥ 830 or 840 nm in both strains (*P* < 0.05, one-way ANOVA and the Tukey *post hoc* honestly significant difference [HSD] test). Thus, unlike the *lws*^+*2a*+*5b*^ medaka, which exhibited the significant attenuation of OMR at much shorter wavelengths (i.e., λ ≥ 740 or 750 nm^25,31^), the behavioural red-light sensitivity of the *lws*^*a:b*^ mutant seems to be equivalent to that of the wild type, despite the copy-number difference.

## Discussion

### Evolutionary history of the medaka *LWS* genes

In this study, we compared four genomic sequences of the *LWS* loci: i.e., *LWSa* and *LWSb* of *O. latipes* and *O. sakaizumii*. The *latipes*–*sakaizumii* speciation was estimated to be about 4–18 million years ago^32,33^. Entire sequences of the *LWS* loci are consistent with the hypothesis that the *LWSa*–*LWSb* duplication preceded this speciation, because the genomic sequences were much more divergent between the duplicates (i.e., paralogues; Fig. 1b) than between the species (i.e., orthologues; Fig. 1a).

However, a part of the *LWS* genes was more similar between the paralogues than between the orthologues (Tables 2 and 3: Fig. 2b). This extraordinary similarity could be explained by non-allelic (ectopic) gene conversion^22^, which seems to frequently occur during the *cone opsin* evolution in vertebrates^34,35^. To explain all the nucleotide substitutions summarized in Tables 1–3, we propose that the sister species could undergo at least two gene conversions, one of which occurred after the *latipes*–*sakaizumii* speciation, for the following reasons.

First, the 13 nucleotides conserved between the paralogues, but not between the orthologues (Tables 2 and 3), could most plausibly be explained by a gene conversion after the speciation. Otherwise, identical substitutions at identical sites of the paralogues (e.g., from C to T substitutions at the 318th nucleotide of *LWSa* and *LWSb* of *O. latipes*) need to occur 13 times, which seems highly unlikely. Second, the region involved in the gene conversion (i.e., from the 3′ half of the 2nd exon to the 6th exon) is much more conserved between the paralogues in *O. latipes* (a total of two substitutions in the 2nd intron) than in *O. sakaizumii* (a total of 17 substitutions [six in the exons and 11 in the introns] and a 1-bp insertion/deletion in the 3rd intron; see also Table 1), indicating that the timing of the latest gene conversion is different between *O. sakaizumii* and *O. latipes* (i.e., earlier in *O. sakaizumii*).

Taken together, one gene conversion occurred very recently in *O. latipes* after the *latipes*–*sakaizumii* speciation. The timing of another gene conversion for *O. sakaizumii* could be close to the timing of the speciation, because the number of nucleotide substitutions is similar between the orthologues and between the paralogues (Table 1; Fig. 2b), but the actual timing could not be specified in this study.

We suspect that even more gene conversion might affect the 1st and the 5′ half of the 2nd exons, if Beloniformes (e.g., medaka and flying fish) and Cyprinodontiformes (e.g., guppy and platy) had shared the same duplication event^4^. This is because; (1) the branch length of the phylogenetic tree in Fig. 2a is apparently shorter in medaka than in guppy (i.e., 6–7 substitutions between the medaka paralogues and 6–25 substitutions between the guppy paralogues), and (2) even this region fails to show orthologous relationships between LWSa/b of medaka and LWS1–4 of guppy (Fig. 2a). Alternatively, these fish might not share the same duplication event (Lin *et al*. [2005] used only *O. latipes* as a representative of Beloniforms^4^). A chance for assessing the true timing of gene duplication has anyway been lost, if gene conversion has involved the entire loci.

Considering that at least two gene conversions could occur during speciation of sister species, non-allelic gene conversion between tandemly duplicated genes would be rather more frequent events than previously supposed^36^. This notification is important, because molecular-phylogenetic analyses alone could mislead the history of gene evolution (i.e., the orthologous/paralogous relationship). Indeed, from the phylogenetic tree using the entire cDNA sequences (Fig. 2c), the medaka *LWS*s seem to undergo very recent and lineage-specific gene duplications, which is highly unlikely according to the present study (Fig. 1; Tables 1–3).

### Functional importance for retaining two nearly identical *LWS* genes

Four paralogous LWSs of guppy have undergone distinct diversification (neo-functionalization) showing divergent λ_max_^20^ (see also Fig. 2). Although the LWSs that medaka currently have are nearly identical in sequence and λ_max_^18^, a trace of such diversification seems to remain as four non-synonymous substitutions between the paralogues in the 1st and 2nd exons (Table 1). Thus, we suspect that λ_max_ of the medaka LWSa and LWSb were previously different, as LWSs of other vertebrates^19–21^. Alternatively, diversification of the medaka LWSs might be suppressed even prior to the conversions (e.g., by reduced mutation rates). Whichever the case is, the following questions need to be addressed. (1) How were the converted alleles fixed in medaka (e.g., by selection or genetic drift)? (2) Why do medaka continue to retain the very similar duplicates, instead of losing either?

After the 3 R whole-genome duplication which occurred at a common ancestor of teleosts, 70–80% of the duplicated genes were lost taking 60 million years, most likely because of functional redundancy^37^. The fact that wild-type medaka show no apparent advantage in red-light sensitivity in comparison with the *lws*^*a:b*^ mutant (Fig. 5) suggests that the paralogues are indeed redundant and either could be dispensable. In addition, the alternative expression system of the *LWS* (and other *cone opsin*) paralogues known in human and zebrafish (i.e., a shared upstream enhancer competitively accessed by the *LWS* promoters^38–40^), which medaka may also have^24^, seems to be nonsense, if the alternates are identical. Considering these discrepancies, we propose that the current *cone opsin* repertoire of medaka is at a provisional state and about to reduce the copy number of *LWS*. Medaka may have retained the duplicates because gene conversion is a quicker and more frequent^34,35^ solution than gene loss for reducing genetic diversity.

However, it is also possible that the alternative expression of the nearly identical (i.e., different) LWSs is not necessary for medaka in the laboratory (Fig. 5), but is in nature. From this standpoint, a recent population genetic study using stickleback, which demonstrated a dramatic change of allele frequency in *SWS2* after 19 years of habituation to a new habitat^41^, is intriguing. Similar *trans*-generation experiments using the wild-type and *lws*^*a:b*^ medaka (and a knock-in medaka with divergent LWSs) would be worthwhile to elucidate which *cone opsin* repertoire could dominate the population. Whatever is the result, however, the process (i.e., whether or not the repertoire actually provides behavioural/ecological advantages to the fish) would remain unknown (i.e., natural/sexual selection vs genetic drift). To reveal the actual driving forces of the *cone opsin* diversifications, establishment and behavioural phenotyping of *cone opsin* mutants, as shown here, would also be necessary.

## Methods

### Ethical issues

All the experiments presented here were conducted in accordance with the Animal Experiment Committees of JWU and NIBB. All the experimental protocols were approved by the same committees.

### Sequence analysis *in silico*

We obtained genomic sequences encompassing the *LWSa* and *LWSb* loci of *O. latipes* (the Hd-rR strain) and *O. sakaizumii* (the HNI strain) from the UTGB database (version 2.2.4; http://utgenome.org/medaka_v2/#!Top.md). We manually annotated the sequences according to the *LWS* sequences in GenBank (*LWSa*: AB223051 and *LWSb*: AB223052) and also by homology search using the BLASTX program. The annotated sequences were manually trimmed (e.g., between the 3′ and 5′ ends of neighbouring genes) and aligned using the LAGAN program at the mVISTA website (http://genome.lbl.gov/vista/mvista/submit.shtml). For more detailed local comparisons, we used the Genetyx-Mac (ver. 16) software.

Phylogenetic reconstruction was performed by the PhyML^42^ at the ATGC website (http://www.atgc-montpellier.fr) using the default settings; i.e., a substitution model was automatically selected by the smart model selection (SMS) based on the Akaike’s information criterion (AIC)^43^, and a starting tree was constructed by the BIONJ^44^. For an outgroup, we used *LWS* of *O. melastigma* (XM_024269264), together with *LWS1–4* of guppy (*Poecilia reticulata*; AB748985, LC127183, LC127184, and LC127185), *LWS* of tilapia (*Oreochromis niloticus*; AF247128), and *LWS1/2* of zebrafish (*Danio rerio*; AB087803 and AB087804).

### Establishment of a medaka strain with a single *LWS* gene; the *lws*^*a:b*^ mutant

As described elsewhere^25^, we microinjected *Cas9* mRNA and gRNA into fertilized eggs of control medaka: the *color interfere* (*ci*) and Actb–SLα:GFP strains^28,29^. *ci* lacks a fish-specific hormone, somatolactin alpha (SLα) and Actb–SLα:GFP overexpresses SLα^28,29^. Neither strain exhibits defect in viability or fertility in comparison with wild type^45^. We have been using these strains for establishing/characterizing colour-blind lines^25,31,46^, because of their unique behaviours showing colour-dependent mate choice^47–49^.

The gRNA targets the 2nd exons of the *LWSa* and *LWSb* loci (5′-GCGTGTTTGAGGGCTATGTGG-3′) which are tightly linked on chromosome 5^15,18^. Whether a deletion of the approximately 7-kb region sandwiched by the target sites occurred was detected by genomic PCR and agarose-gel electrophoresis using two combinations of three primers: *LWSa*-specific forward: 5′-GGCAGAAAAGTTGGTTGGAT–3′, *LWSb*-specific forward: 5′-TTGTTTCCCAGATCCCTTTG-3′, and *LWSb*-specific reverse: 5′-CAATTCTAGTGATTCAAGACTCATTTATAAAAG-3′, whose positions are shown in Fig. 3a (see figure legend for the rational). PCR conditions were 60 s at 94 °C for initial denaturation, 40 cycles of 20 s at 98 °C, 1 min at 60 °C, 1 min at 72 °C, and 10 min at 72 °C for final extension. The amplified products in the gel were visualized by ethidium-bromide staining and UV irradiation.

### Semi-quantitative reverse transcription PCR

We extracted total RNA from the eyes of adult medaka (under 1 year old) using ISOGEN II (Nippon Gene). The RNA was treated with deoxyribonuclease (RT Grade) for Heat Stop (Nippon Gene) and used for reverse transcription (RT) using ReverTra Ace (TOYOBO) and polyT primer. Using the RT products (cDNAs) as templates, we performed PCR using the following primers, which were designed to commonly amplify the *LWSa*, *LWSb*, and hybrid *LWSa:b* cDNAs; forward: 5′-GGCAGAGSAGTGGGGAAAACAGG-3′ and reverse: 5′-TATGCAGGAGCCACAGAGGAGACC-3′. For a control, we amplified the *β-actin* (*Actb*) cDNA using the following primers; forward: 5′-GATTCCCTTGAAACGAAAAGCC-3′ and reverse: 5′-CAGGGCTGTTGAAAGTCTCAAAC-3′. PCR conditions were 60 s at 94 °C for initial denaturation, 16–26 cycles of 20 s at 98 °C, 1 min at 58 °C, 1 min at 72 °C, and 10 min at 72 °C for final extension. All the products were visualized by agarose-gel electrophoresis, ethidium-bromide staining, and UV irradiation.

### Optomotor response (OMR) test using the Okazaki large spectrograph (OLS)

As previously described^31^, we performed quantitative OMR tests using the *lws*^*a:b*^ mutants (n = 6), which we outline here briefly.

The OLS generates a series of monochromatic lights (λ = 250–1,000 nm) of about 10-m width using a xenon arc lamp and a diffraction grating^50^. The light intensity (photon density) varies depending on wavelength, but was 1.4–7.5 × 10^15^ photons/cm^2^/s at λ = 720–850 nm^31^. Using light at an intended wavelength, we illuminated from the top a cylindrical glass tank and an electronic apparatus for rotating black-and-white stripes around the tank. We placed individual medaka into the tank, started the OLS illumination, waited for 30 s, and rotated the stripes in a clockwise, counter-clockwise, and clockwise direction for 30 s each for an OMR test. The behaviour of the fish was recorded using an infra-red (IR)-sensitive video camera (A10FHDIR; Kenko) and the position of the fish in each frame (x–y coordinates) was detected by the UMATracker software^51^.

Using the series of x–y coordinates, we calculated (1) the average time (s) in the three rotations until the fish started the OMR (delay), (2) the average period (%) of the fish showing the OMR in the tree rotations (duration), and (3) the total distance (round) the fish swam towards the direction of the rotating stripes during the test (distance). The data were averaged at each wavelength and compared between the wild type and *lws*^*a:b*^ mutant by Student’s *t* test. We also compared the averages in each strain at different wavelengths using one-way analysis of variance (ANOVA) and the Tukey *post hoc* HSD test. The data for the wild-type fish (n = 5) in Fig. 5 are those previously reported^31^.

## Supplementary information

SupplementaryFigures

## References

[1] Bowmaker JK (1998). Evolution of colour vision in vertebrates. Eye.

[2] Rennison DJ, Owens GL, Taylor JS (2012). Opsin gene duplication and divergence in ray-finned fish. Mol. Phylogenet. Evol..

[3] Bowmaker JK (2008). Evolution of vertebrate visual pigments. Vision Res..

[4] Lin JJ, Wang FY, Li WH, Wang TY (2017). The rises and falls of opsin genes in 59 ray-finned fish genomes and their implications for environmental adaptation. Sci. Rep..

[5] Valen, R., Edvardsen, R. B., Søviknes, A. M., Drivenes, Ø. & Helvik, J. V. Molecular evidence that only two opsin subfamilies, the blue light- (SWS2) and green light-sensitive (RH2), drive color vision in Atlantic Cod (Gadus morhua). *PLoS One*, 10.1371/journal.pone.0115436 (2014). 10.1371/journal.pone.0115436 PMC4281148 25551396

[6] Escobar-Camacho D, Ramos E, Martins C, Carleton KL (2017). The opsin genes of amazonian cichlids. Mol. Ecol..

[7] Wang, F. Y., Chung, W. S., Yan, H. Y. & Tzeng, C. S. Adaptive evolution of cone opsin genes in two colorful cyprinids, Opsariichthys pachycephalus and Candidia barbatus. *Vision Res*. 10.1016/j.visres.2008.04.026 (2008). 10.1016/j.visres.2008.04.026 18571688

[8] Cole BL (2002). Protan colour vision deficiency and road accidents. Clin. Exp. Optom..

[9] Melin AD (2017). Trichromacy increases fruit intake rates of wild capuchins (*Cebus capucinus imitator*). Proc. Natl. Acad. Sci..

[10] Veilleux CC (2016). Group benefit associated with polymorphic trichromacy in a Malagasy primate (Propithecus verreauxi). Sci. Rep..

[11] Hogan JD, Fedigan LM, Hiramatsu C, Kawamura S, Melin AD (2018). Trichromatic perception of flower colour improves resource detection among New World monkeys. Sci. Rep..

[12] Mokodongan DF (2018). Phylogenomics reveals habitat-associated body shape divergence in Oryzias woworae species group (Teleostei: Adrianichthyidae). Mol. Phylogenet. Evol..

[13] Asai T, Senou H, Hosoya K (2011). Oryzias sakaizumii, a new ricefish from northern Japan (Teleostei: Adrianichthyidae). Ichthyol. Explor. Freshwaters.

[14] Kasahara M (2007). The medaka draft genome and insights into vertebrate genome evolution. Nature.

[15] Naruse K (2000). A detailed linkage map of medaka, Oryzias latipes: comparative genomics and genome evolution. Genetics.

[16] Fukamachi S, Shimada A, Shima A (2001). Mutations in the gene encoding B, a novel transporter protein, reduce melanin content in medaka. Nat. Genet..

[17] Matsuda M, Sakaizumi M (2016). Evolution of the sex-determining gene in the teleostean genus Oryzias. Gen. Comp. Endocrinol..

[18] Matsumoto Y, Fukamachi S, Mitani H, Kawamura S (2006). Functional characterization of visual opsin repertoire in Medaka (Oryzias latipes). Gene.

[19] Chinen A, Hamaoka T, Yamada Y, Kawamura S (2003). Gene duplication and spectral diversification of cone visual pigments of zebrafish. Genetics.

[20] Kawamura S (2016). Spectral sensitivity of guppy visual pigments reconstituted *in vitro* to resolve association of opsins with cone cell types. Vision Res..

[21] Kawamura S (2016). Color vision diversity and significance in primates inferred from genetic and field studies. Genes and Genomics.

[22] Hastings PJ (2010). Mechanisms of Ectopic Gene conversion. Genes (Basel)..

[23] Ewen-Campen, B., Mohr, S. E., Hu, Y. & Perrimon, N. Accessing the Phenotype Gap: Enabling Systematic Investigation of Paralog Functional Complexity with CRISPR. *Developmental Cell*, 10.1016/j.devcel.2017.09.020 (2017). 10.1016/j.devcel.2017.09.020 29017030

[24] Daido Y, Hamanishi S, Kusakabe TG (2014). Transcriptional co-regulation of evolutionarily conserved microRNA/cone opsin gene pairs: Implications for photoreceptor subtype specification. Dev. Biol..

[25] Homma N, Harada Y, Uchikawa T, Kamei Y, Fukamachi S (2017). Protanopia (red color-blindness) in medaka: A simple system for producing color-blind fish and testing their spectral sensitivity. BMC Genet..

[26] Hsu PD (2013). DNA targeting specificity of RNA-guided Cas9 nucleases. Nat. Biotechnol..

[27] Yokoyama S, Yang H, Starmer WT (2008). Molecular basis of spectral tuning in the red- and green-sensitive (M/LWS) pigments in vertebrates. Genetics.

[28] Fukamachi S, Sugimoto M, Mitani H, Shima A (2004). Somatolactin selectively regulates proliferation and morphogenesis of neural-crest derived pigment cells in medaka. Proc. Natl. Acad. Sci..

[29] Fukamachi S, Yada T, Meyer A, Kinoshita M (2009). Effects of constitutive expression of somatolactin alpha on skin pigmentation in medaka. Gene.

[30] Takehana Y (2016). Origin of Boundary Populations in Medaka (*Oryzias latipes* Species Complex). Zoolog. Sci..

[31] Matsuo M, Ando Y, Kamei Y, Fukamachi S (2018). A semi-automatic and quantitative method to evaluate behavioral photosensitivity in animals based on the optomotor response (OMR). Biol. Open.

[32] Takehana Y, Nagai N, Matsuda M, Tsuchiya K, Sakaizumi M (2003). Geographic Variation and Diversity of the Cytochrome *b* Gene in Japanese Wild Populations of Medaka, Oryzias latipes. Zoolog. Sci..

[33] Setiamarga DHE (2009). Divergence time of the two regional medaka populations in Japan as a new time scale for comparative genomics of vertebrates. Biol. Lett..

[34] Hiwatashi T (2011). Gene conversion and purifying selection shape nucleotide variation in gibbon L/M opsin genes. BMC Evol. Biol..

[35] Cortesi F (2015). Ancestral duplications and highly dynamic opsin gene evolution in percomorph fishes. Proc. Natl. Acad. Sci..

[36] Harpak A, Lan X, Gao Z, Pritchard JK (2017). Frequent nonallelic gene conversion on the human lineage and its effect on the divergence of gene duplicates. Proc. Natl. Acad. Sci..

[37] Inoue, J., Sato, Y., Sinclair, R., Tsukamoto, K. & Nishida, M. Rapid genome reshaping by multiple-gene loss after whole-genome duplication in teleost fish suggested by mathematical modeling. *Proc. Natl. Acad. Sci*. 10.1073/pnas.1507669112 (2015). 10.1073/pnas.1507669112 PMC4672829 26578810

[38] Takechi M (2005). Temporal and spatial changes in the expression pattern of multiple red and green subtype opsin genes during zebrafish development. J. Exp. Biol..

[39] Tsujimura T, Hosoya T, Kawamura S (2010). A single enhancer regulating the differential expression of duplicated red-sensitive opsin genes in Zebrafish. PLoS Genet..

[40] Tsujimura T, Chinen A, Kawamura S (2007). Identification of a locus control region for quadruplicated green-sensitive opsin genes in zebrafish. Proc. Natl. Acad. Sci..

[41] Marques DA (2017). Convergent evolution of SWS2 opsin facilitates adaptive radiation of threespine stickleback into different light environments. PLoS Biol..

[42] Guindon S (2010). New algorithms and methods to estimate maximum-likelihood phylogenies: Assessing the performance of PhyML 3.0. Syst. Biol..

[43] Lefort, V., Longueville, J. E. & Gascuel, O. SMS: Smart Model Selection in PhyML. *Mol. Biol. Evol*., 10.1093/molbev/msx149 (2017). 10.1093/molbev/msx149 PMC5850602 28472384

[44] Gascuel O (1997). BIONJ: An improved version of the NJ algorithm based on a simple model of sequence data. Mol. Biol. Evol..

[45] Komine R (2016). Transgenic medaka that overexpress growth hormone have a skin color that does not indicate the activation or inhibition of somatolactin-α signal. Gene.

[46] Kamijo M, Kawamura M, Fukamachi S (2018). Loss of red opsin genes relaxes sexual isolation between skin-colour variants of medaka. Behav. Processes.

[47] Fukamachi S (2009). Dual control by a single gene of secondary sexual characters and mating preferences in medaka. BMC Biol..

[48] Ikawa M, Ohya E, Shimada H, Kamijo M, Fukamachi S (2017). Establishment and maintenance of sexual preferences that cause a reproductive isolation between medaka strains in close association. Biol. Open.

[49] Utagawa U, Higashi S, Kamei Y, Fukamachi S (2016). Characterization of assortative mating in medaka: Mate discrimination cues and factors that bias sexual preference. Horm. Behav..

[50] Watanabe M (1982). Design and performance of the Okazaki Large Spectrograph for photobiological research. Photochem Photobiol.

[51] Yamanaka, O. & Takeuchi, R. UMATracker: an intuitive image-based tracking platform. *J. Exp. Biol*. jeb. 182469, 10.1242/jeb.182469 (2018). 10.1242/jeb.182469 29954834

