# Supplementary material for: Evolutionary history of the medaka long-wavelength sensitive genes and effects of artificial regression by gene loss on behavioural photosensitivity
Source: Sci Rep. 2019 Feb 25;9:2726. doi: 10.1038/s41598-019-39978-6 (PMC6389941; doi:10.1038/s41598-019-39978-6)
Supplement: Supplementary file 1 — SupplementaryFigures [file 41598_2019_39978_MOESM1_ESM.pdf]

Supplementary Figures for;

Evolutionary history of the medaka long-wavelength sensitive genes and effects of artificial regression by gene loss on behavioural photosensitivity

Yumi Harada, Megumi Matsuo, Yasuhiro Kamei, Mayuko Goto, & Shoji Fukamachi

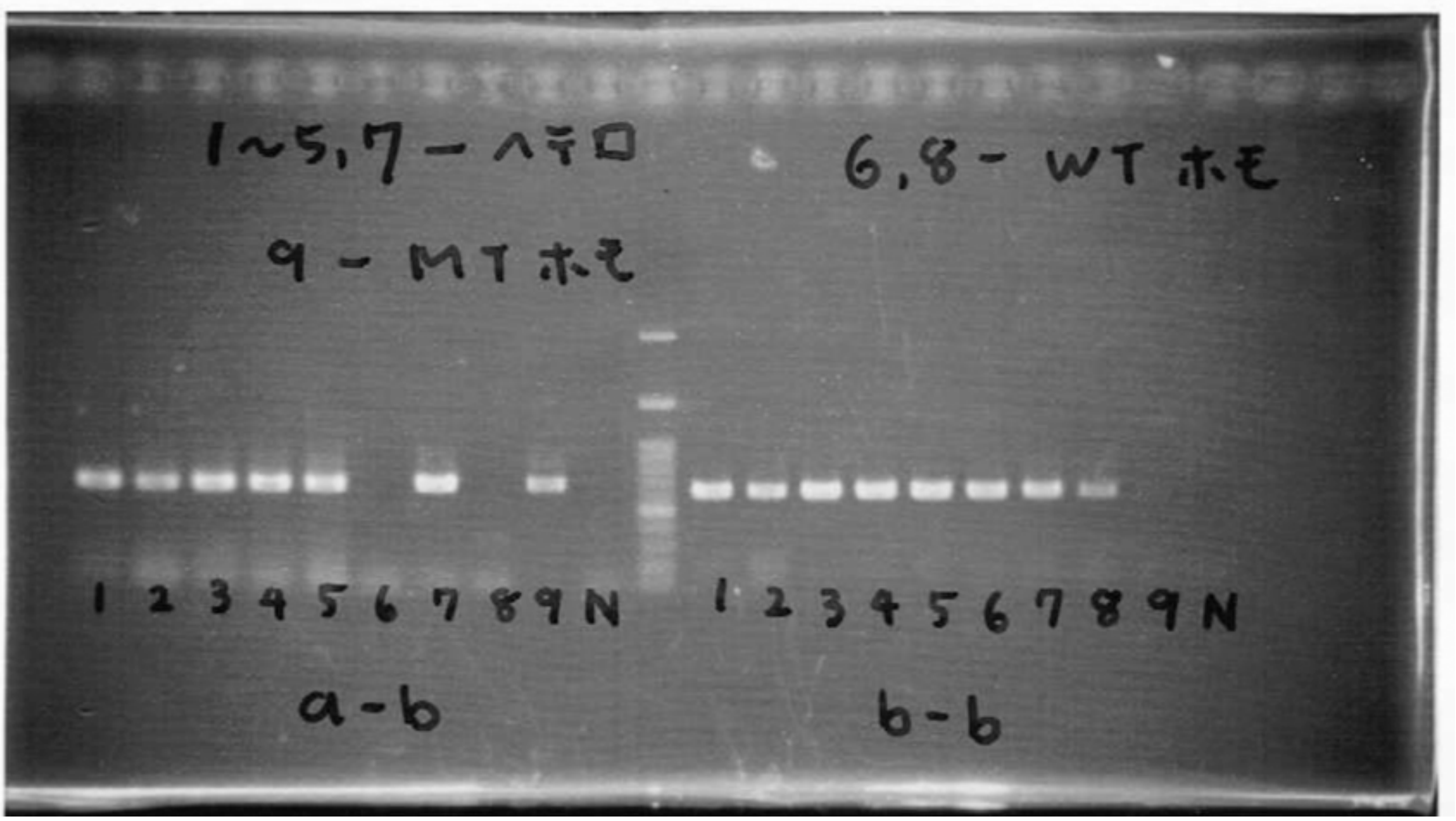

Supplementary Figure 1. An original gel image for Figure 3B.

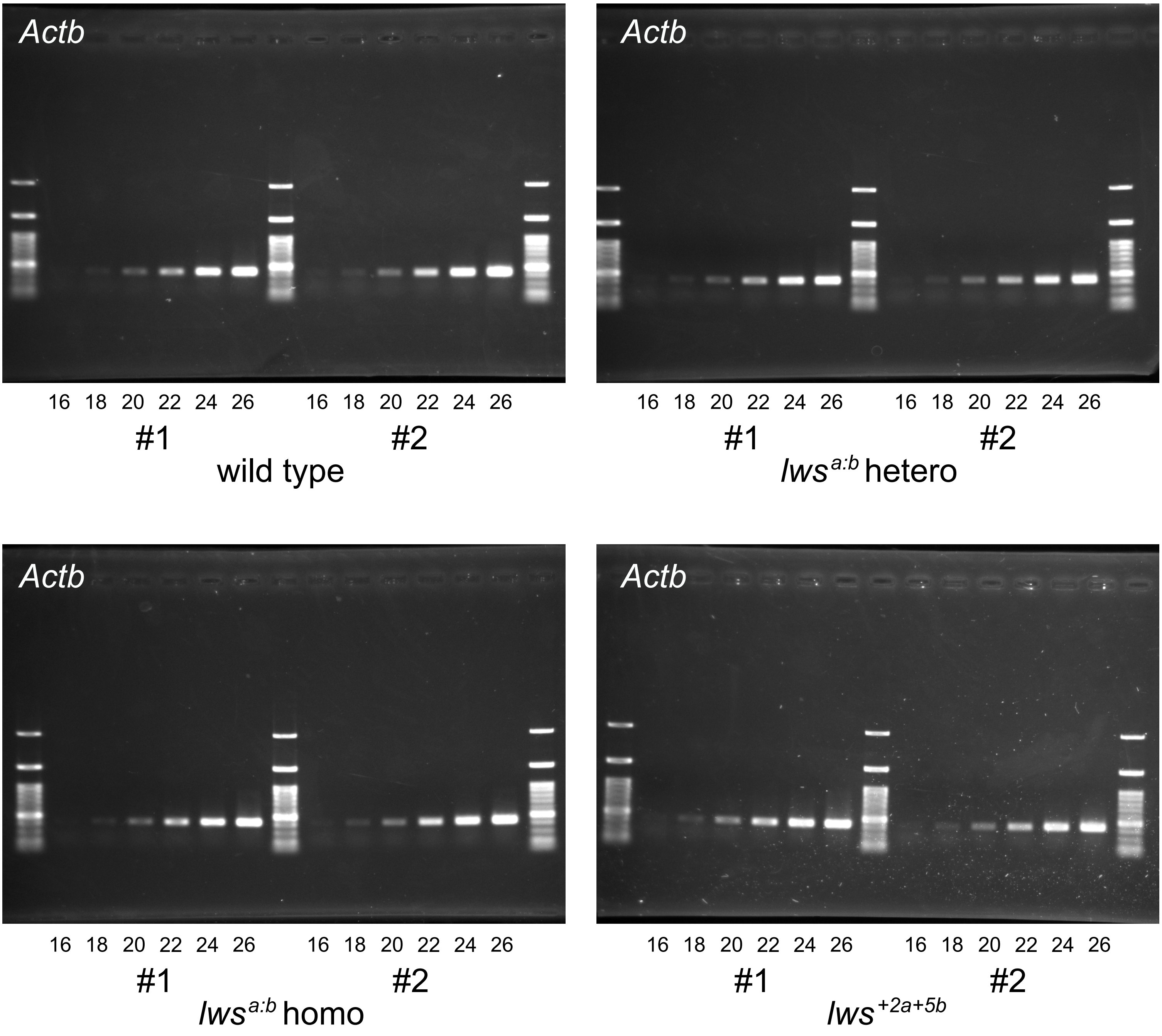

Supplementary Figure 2. Semi-quantitative RT-PCR of the *Actb* and *LWSa/b/a:b* genes. The number of PCR cycles (16-26), fish ID (#1 & #2), and genotypes for the *LWS* loci are shown. The products electrophorized in Figure 4A are those at 24 cycles.

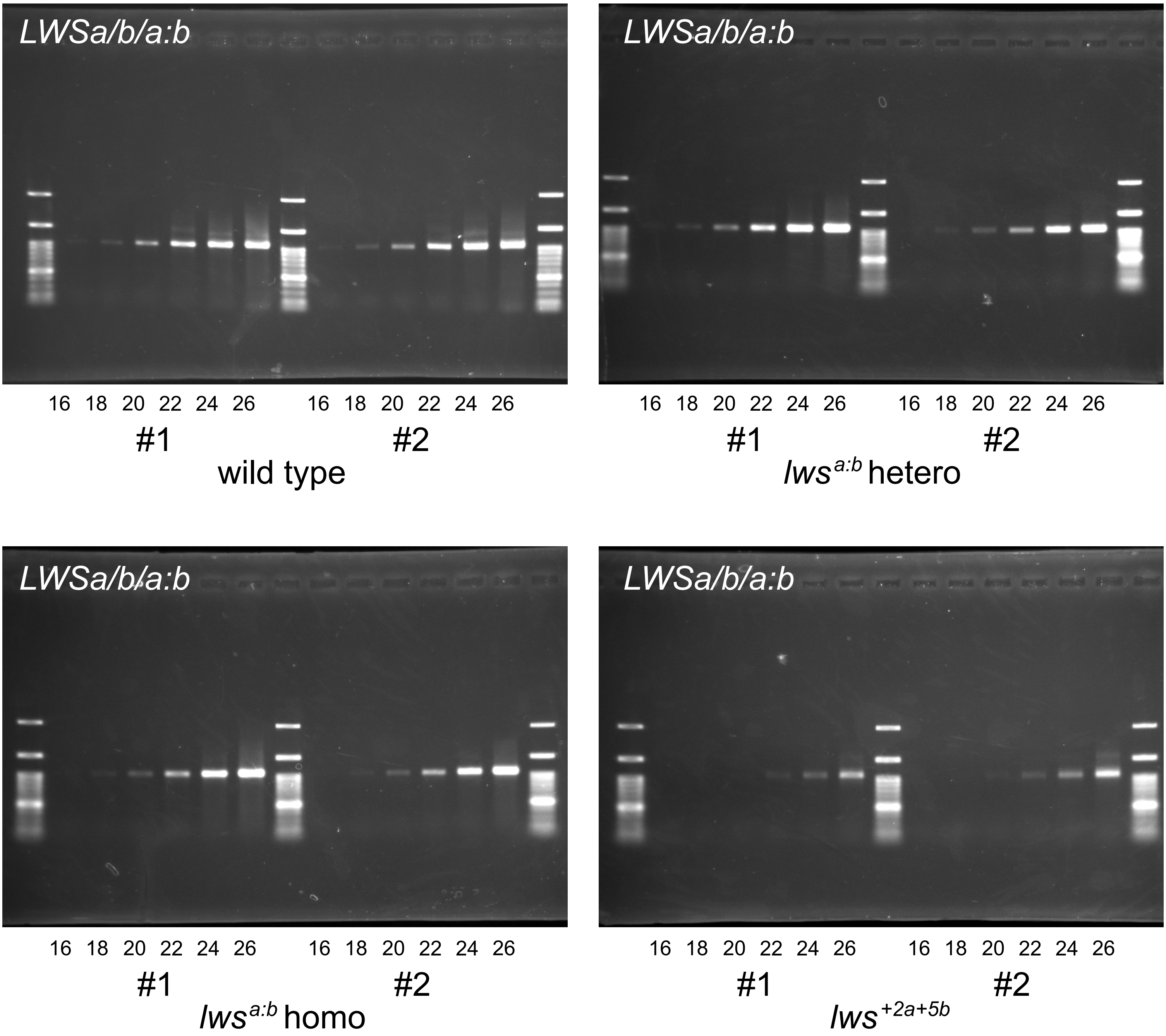

Supplementary Figure 2. (continued)
